# Endoplasmic Reticulum-Mediated Protein Quality Control and Endoplasmic Reticulum-Associated Degradation Pathway Explain the Reduction of N-glycoprotein Level Under the Lead Stress

**DOI:** 10.3389/fpls.2020.598552

**Published:** 2021-01-13

**Authors:** Hong Du, Canqi Zheng, Muhmmad Aslam, Xihui Xie, Wanna Wang, Yingquan Yang, Xiaojuan Liu

**Affiliations:** ^1^Guangdong Provincial Key Laboratory of Marine Biotechnology, STU-UNIVPM Joint Algal Research Center, College of Sciences, Institute of Marine Sciences, Shantou University, Shantou, China; ^2^Southern Marine Science and Engineering Guangdong Laboratory, Guangzhou, China; ^3^Faculty of Marine Sciences, Lasbela University of Agriculture, Water & Marine Sciences, Uthal, Pakistan

**Keywords:** protein level, lead, N-glycan, ER-mediated protein quality control, ER-associated degradation pathway

## Abstract

Different anthropogenic activities result in the continuous increase of metal lead (Pb) in the environment and adversely affect living organisms. Therefore, it is important to investigate the tolerance mechanism in a model organism. *Chlamydomonas reinhardtii* is an important green eukaryotic model microalga for studying different kinds of biological questions. In this study, the responses of *C. reinhardtii* were revealed via a comprehensive approach, including physiological, genomic, transcriptomic, glycomic, and bioinformatic techniques. Physiological results showed that the growth rate and soluble protein content were significantly reduced under the high lead stress. Also, the results obtained from the genomic and transcriptomic analyses presented that the endoplasmic reticulum-mediated protein quality control (ERQC) system and endoplasmic reticulum-associated degradation (ERAD) pathway were activated under the third day of high lead stress. The unique upregulated protein disulfide isomerase genes on the ERQC system were proposed to be important for the protein level and protein quality control. The accumulation of specific N-glycans indicated that specific N-glycosylation of proteins might alter the biological functions of proteins to alleviate the Pb stress in alga and/or lead to the degradation of incomplete/misfolded proteins. At the same time, it was observed that genes involved in each process of ERAD were upregulated, suggesting that the ERAD pathway was activated to assist the degradation of incomplete/misfolded proteins. Therefore, it is reasonable to speculate that the reduction of protein level under the high lead stress was related to the activated ERQC system and QRAD pathway. Our findings will provide a solid and reliable foundation and a proposed ERAD working model for further in-depth study of the ERQC system and ERAD pathway under the Pb stress and even other biotic and abiotic stresses.

## Introduction

Metal pollution is one of the most serious global environmental problems threatening algae, plants, animals, and even human beings (Peharec Štefanić et al., 2012). Among all metals, lead (Pb) is one of the most toxic metals, with a long half-life. When Pb was released into the environment, algae and plants were physiologically affected, particularly in terms of the inhibition of growth and photosynthesis (Du et al., 2018), the increased production of reactive oxygen species (Szivák et al., 2009), and the changed phytochelatin production and kinetics, metabolism, and ultrastructure of cells (Sharma and Dubey, 2005), and the damage of lipid, protein, and nucleic acid (Hasan et al., 2017). In addition, gene expression and protein translation were also affected by Pb in algae and plants via transcriptome and proteome analysis (Wang et al., 2013; Li et al., 2016). Transcriptome results implied that differentially expressed genes involved in the cell wall and glutathione metabolism were upregulated, but genes related to carbohydrate metabolism were downregulated during Pb stress (Wang et al., 2013). Proteome analysis showed that proteins involved in signal transduction, redox, stress, and transport were upregulated, whereas proteins related to nucleotide, energy, protein, and carbohydrate metabolisms were downregulated under the lead stress (Li et al., 2016; Du et al., 2018). The inconsistency between transcript and protein levels suggested that both transcriptional and translational regulations played important roles in response to Pb contamination (Li et al., 2016).

Nascent translated proteins in response to stresses were largely dependent on co-/post-translational modifications (e.g., N-glycosylation) that altered the properties and biological functions of proteins, including activity, localization, and/or interaction with other proteins and consequently affected the phenotype of organisms (Peharec Štefanić et al., 2012). N-glycosylation happens in the endoplasmic reticulum, and the Golgi apparatus is a major co-/post-translational modification in the secretory pathway of proteins. Suitable complex N-glycan formation and processing regulate glycoproteins for a plant to deal with different stresses. For example, it was documented that mannosidase-mediated trimming of N-glycans was important for *Arabidopsis* tolerance to salt and osmotic stresses (Liu et al., 2018). Xylose residues on N-glycans created a regulatory role for seed germination, rice growth, and development under phytohormone treatments and other abiotic stresses, including temperature, drought, salinity, and oxygen-deficiency stresses (Takano et al., 2015). Additionally, missing core fucose residues but not xylose in N-glycans strengthened salt sensitivity of *Arabidopsis* hybrid glycosylation mutant (Kaulfürst-Soboll et al., 2011). On the contrary, some N-glycans were proved to be critical for mature N-glycosylation of proteins but not essential for plants’ defense under abiotic stresses. For example, 1,3-mannosyltransferase-dependent N-glycans did not affect *Arabidopsis* phenotype under normal and high temperature or sale/osmotic stress conditions (Kajiura et al., 2010). Therefore, the structure of N-glycans played an important role in protein folding and quality control and defense under different stresses. However, so far, a complete gap exists in the role of N-glycans formation and protein N-glycosylation in the responses of algae to biotic and abiotic stresses, especially Pb stress.

Nascent glycopeptides are initially folded to their correct conformation in the ER. This process is aided by a known endoplasmic reticulum (ER)-mediated protein quality control (ERQC) mechanism (Blanco-Herrera et al., 2015). Firstly, two glucose residues on the N-glycans of nascent glycopeptides are sequentially removed by glucosidases I and II (GI and GII), producing mono-glucosylated N-glycans. The trimmed N-glycan structures are further recognized by the ER lectin chaperones calnexin (CNX) and/or calreticulin (CRT). Meanwhile, some other ER chaperones and folding enzymes [e.g., protein disulfide isomerases (PDIs)] are also recruited by CNX/CRT for high-specificity high-affinity binding with the mono-glucosylated N-glycans (Nagashima et al., 2018). Subsequently, the remaining glucose residue is removed by GII, the nascent glycoprotein is released from CNX/CRT, and the folding process is finished. Finally, correctly folded glycoproteins will be exported to the Golgi apparatus for further modification and transported to final destinations. However, incomplete/misfolded glycoproteins are recognized and re-glucosylated by an ER-resident folding sensor UDP-glucose: glycoprotein glucosyltransferase (UGGT) for reassociation with CNX/CRT (Caramelo and Parodi, 2008). One or more CNX/CRT cycles are performed until incomplete/misfolded glycoproteins achieving their native conformations. If the glycoproteins that fail to fold its native structure after multiple CNX/CRT cycles are removed from the ERQC system and degraded by an ER-associated degradation pathway (ERAD) (Shin et al., 2018). ERAD mainly contains three steps: (1) the marking of terminally misfolded glycoproteins, (2) recruitment, retrotranslocation, and ubiquitination of misfolded glycoproteins, and (3) extraction, processing, and delivery of misfolded glycoproteins to the proteasome for final degradation. It was known that ERQC is an environmental sensor and responder in plants under different stresses. When the demands for protein folding exceed the capacity of ERQC under stress, the ER protein folding mechanism reaches its limit (Liu and Li, 2014). In this situation, incomplete/misfolded glycoproteins are largely accumulated in the ER, stimulating an unfolded protein response (UPR) (Liu and Howell, 2010). Subsequently, UPR will alleviate ER stress via upregulating the expression of genes involved in ERQC and/or ERAD. For example, the NtPDI gene involved in ERQC was upregulated under the Cd stress in *Nicotiana tabacum* (Xu et al., 2013). AtHRD1 (a gene associated with ERAD) mutant was more sensitive to Se stress in *Arabidopsis* (Van Hoewyk, 2013). Therefore, it is curious to study the ERQC and ERAD mechanisms under the lead stress and see how the ERQC and ERAD affect the protein level and quality control.

*Chlamydomonas reinhardtii* is a model green alga that is widely used to study flagellar biogenesis, circadian rhythms, photosynthesis, responses to nutrient deficiency, and so on (Merchant et al., 2007; Harris, 2013). So far, the responses of *C. reinhardtii* to Pb stress were also studied, such as the role of phytochelatin in Pb detoxification (Scheidegger et al., 2011), bioaccumulation and biosorption of Pb (Stewart et al., 2015), and its compartmentalization in vacuoles (Sánchez-Marín et al., 2014). Furthermore, it was documented that exposure to metals led to changes in the expression of proteins in *C. reinhardtii* (Gillet et al., 2006; Walliwalagedara et al., 2012). However, it is still unknown how the Pb stress changes protein folding and quality control and the effects of the ERQC system and ERAD in *C. reinhardtii* under the Pb stress.

In this study, a comprehensive approach, including physiological, genomic, transcriptomic, glycomic, and bioinformatic analyses, was carried out to investigate the physiological effects, especially the protein content, and the ERQC and ERAD occurring in *C. reinhardtii* under Pb stress. Results revealed that the growth was significantly inhibited; soluble protein content was extremely reduced after the third day of high lead stress. N-glycan structures of glycoproteins were changed, indicating their importance for the protein level and protein quality control. Additionally, it was speculated that the ERQC system and QRAD were activated to modify and/or remove incomplete/misfolded proteins to maintain ER homeostasis under the third day of high lead stress. Therefore, it was concluded that the reduction of protein content might be owing to the activated ERQC system and ERAD pathway. Our findings will provide an important opportunity to advance the understanding of the effects of the ERQC system and ERAD pathway on the protein level and quality control under the Pb stress.

## Materials and Methods

### Microalgal Strain and Culture Conditions

*Chlamydomonas reinhardtii* (CC-503 cw903 mt+) from Freshwater Algae Culture Collection (Institute of Hydrobiology, Wuhan, China) was used in this study. Algae were firstly preincubated in Tris-acetate-phosphate medium under the condition of 22°C, 12-h light:12-h darkness photoperiod, 0.08 mol photons m^–2^ s^–1^ light intensity in 100 rpm rotary shaker (Hutner, 1990). During the mid-exponential period of algal growth, cells were cultured in fresh modified Tris-acetate-phosphate medium (1.0 × 10^5^ cells ml^–1^), where the KH_2_PO_4_ was substituted for Na_2_-glycerol-2-phosphate (Kopittke et al., 2008). pH was controlled to around 6 (Hassler et al., 2004). Ten millimolar 2-(N-morpholino) ethane sulfonate (MES, sodium salt, Sigma) medium was used as a pH buffer. Finally, algal mediums containing 0, 3, and 80 μmol L^–1^ Pb(NO_3_)_2_ were prepared. Nitrate was used as a counter ion, as its low possibility to form metal complexes. Nitric acid was applied to the adjustment of the pH value. All experiments were performed in triplicates.

### Measurements of Cell Density and Soluble Protein Content

On 1/2, 1, 3, 5, and 7 days, cell density of *C. reinhardtii* was analyzed by an electronic particle counter (Orifice, 50 μm; Multisizer II; Beckham Coulter, Fullerton, CA, United States) (Zheng et al., 2013).

On the third and seventh days, 40-ml *C. reinhardtii* culture was centrifuged at 3,000 × *g* for 10 min. Concentrated *C. reinhardtii* cells were resuspended in 1 ml of 20-mM phosphate-buffered saline buffer (pH 7.4) and 10-μl 25 × protease inhibitor cocktail and sonicated on 3 s/off 5 s for 3 min, 90 W. After centrifugation at 12,000 × *g* for 20 min, 50-μl aliquot of the supernatant was used to determine the soluble protein concentration with Coomassie brilliant blue kit (Jiancheng Biotech Company, Nanjing, China) via UV-VIS spectrophotometry at 595 nm. To purify protein, the remaining supernatant was exhaustively dialyzed against 2 L of water at 4°C for 48 h. During the process of dialysis, water was refreshed every 8 h. All the steps for protein preparation were carried out at 4°C. The dialyzed samples were lyophilized for the next experiments.

### Release of N-Glycans With PNGase F and *in situ* Derivatization With Fmoc

On the third day, 40-ml algal cells were harvested for the extraction of total glycoprotein. The release and derivatization of N-glycans from protein samples were mainly performed as described in previous article (Kamoda et al., 2005; Nakano et al., 2009). Briefly, 10 μg of each glycoprotein sample was completely dissolved in 100 μl of 20-mM phosphate buffer (pH 8.5). One microliter PNGase F (one unit) was added to the glycoprotein sample and incubated at 37°C for 2 h to release N-glycans. Subsequently, the mixture was added 300-μl deionized water for dilution and 200-μl fresh solution of Fmoc-Cl in acetone (50 mg ml^–1^) and incubated at 37°C for 2 h to derive the N-glycans. Afterward, to remove excess Fmoc reagent, 300-μl chloroform was used to wash N-glycans. The washing steps were repeated at least five times. Finally, the chloroform layer was removed, and the aqueous layer containing Fmoc-linked N-glycans of each sample was dried by an evaporator and dissolved in 20-μl double distilled water. Five microliters was used for the electrospray ionization mass spectrometry (ESI-MS) experiment.

### Electrospray Ionization Mass Spectrometry Conditions

The acquisition range was m/z 1,200–1,900. The mass spectrometer was calibrated via a tune mix (Thermo Scientific). Mass spectra were viewed and analyzed via software Xcalibur V2.2. Additionally, Glycoworkbench software was used to annotate and visualize the structures of N-glycans as described in previous article (Pedersen et al., 2017; Lucas et al., 2018, 2019).

The relative abundance of the different N-glycans under lead stresses was based on the signal intensities of the corresponding Fmoc-labeled N-glycans obtained from ESI-MS analysis as previously described (Nakano et al., 2009; Mathieu-rivet et al., 2013). Briefly, each signal intensity of N-glycans was used to calculate their relative abundance compared with the total signal intensity of all identified N-glycans.

### *In silico* Genome Analysis

On the third day, algal cells under the control and the lead stresses were used to do transcriptomic analysis. The transcriptomic data were referenced from our recent article (Zheng et al., 2020). The sequence reads had already been available by the National Center for Biotechnology Information sequence read archives (SRR10269729, SRR10269730, SRR10269727, SRR10269728, SRR10269731, and SRR10269732). Genes with | log2 fold change| ≥ 1.5 and FDR < 0.01 (adjusted *P*-value, determined by the Benjamini and Hochberg multiple-testing correction implemented in the “p.adjust” method of R) were defined as differentially expressed genes.

Protein sequences involved in the ERQC system and ERAD pathway of *Arabidopsis thaliana* were retrieved from Phytozome 12^1^. The Hidden Markov model (HMM) files corresponding to different domains were downloaded from the Pfam protein family database^2^ against protein sequences from *A. thaliana*. HMMER 3.0 was used to search the genes involved in the ERQC system and ERAD pathway from the *C. reinhardtii* genome database. From the proteins obtained using the HMM, *E*-value < 1e-20 was set to isolate high-quality proteins. All candidate genes based on HMMER results were further manually analyzed by confirming the existence of the core sequences using the SMART program^3^ and National Center for Biotechnology Information Conserved Domains Database^4^.

The genome database of green algae *C. reinhardtii*, *Ostreococcus lucimarinus*, *Chlorella variabilis*, and plant *A. thaliana* were downloaded from Ensembl^5^. Full length and PDI domain sequences of all PDI proteins from algae *C. reinhardtii*, *O. lucimarinus*, *C. variabilis*, and plant *A. thaliana* were aligned using ClustalW2, and a phylogenetic tree was produced using MEGA 6.0 via the neighbor-joining method, with 1,000 replicates of bootstrap analysis. The phylogenetic tree of PDI domains was modified and improved by EvolView. Motifs of all PDI proteins were predicted by the motif elicitation program (MEME)^6^. InterPro database was used for the second identification of conserved motifs^7^. Transmembrane domains of proteins were compared among different servers, including genome database, TOPCONS^8^, TMHMM Server v. 2.0^9^, ΔG predictor^10^, and SOSUI. Putative N-terminal signal peptide was predicted using SignalP 3.0^11^ using default settings. PredictProtein was used to predict the secondary structure^12^.

### Data Statistical Analysis

All the data from experiments were shown as mean ± standard deviation. Data were analyzed using SPSS 20.0 software with one-way ANOVA. The statistical significance was considered at ^∗^*P* < 0.05 for significant and ^∗^*P* < 0.01 for highly significant values. All figures were generated by Origin 8.0 and Adobe Illustrator CS6.

## Results

### Cell Density and Soluble Protein Content of *C. reinhardtii* Under the Lead Stress

**FIGURE 1:**
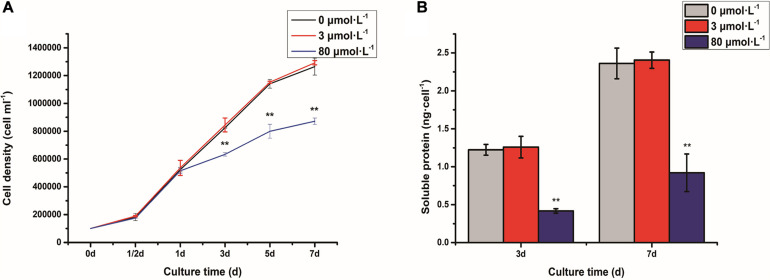
Cell density **(A)** and soluble protein content **(B)** of *Chlamydomonas reinhardtii* under the lead stress. **P < 0.01 representing extremely significant differences in mean value between different stresses. Error bars indicate SD for three biological replicates. Cell density was referenced from our recent article (Zheng et al., 2020).

The cell density of *C. reinhardtii* was measured under the lead stress (Figure 1A). It was shown that the cell density did not have a significant difference between control and low lead stress. However, the cell number was extremely dramatically decreased after 3 days of high lead stress (*P* < 0.01). Soluble protein content was shown in Figure 1B. Obviously, soluble protein content was significantly reduced on the third and seventh days under the high lead stress compared with that under the control and low lead stress (*P* < 0.01).

### Endoplasmic Reticulum-Mediated Protein Quality Control System in *C. reinhardtii* Under Lead Stresses

Homology searches based on sequence similarities with ER-resident proteins in the N-glycosylation pathway were carried out in the genome database of *C. reinhardtii*. The results were presented in Table 1. The N-glycosylation pathway of protein in eukaryotes firstly takes place in the cytosolic side and lumen of the ER. Many glycosyltransferases and glucosidases are involved in the N-glycosylation pathway in the ER, such as ALG, RTF, CNX, CRT, RPN, and STT3. In this study, only four genes related to the N-glycosylation pathway (ALG14, RPN1, RPN2, and STT3B) were differentially expressed under the third day of high lead stress (Table 1). Among these four genes ALG14, RPN2, and STT3B were upregulated, RPN1 was downregulated under the high lead stress compared with that in the control and the low lead stress.

**TABLE 1:** Expression of putative genes involved in the ER protein N-glycosylation of *Chlamydomonas reinhardtii* under the third day of lead stress.

Gene ID	Abbreviation	Domain	FPKM			DESeq_log2FC	
			0	3	80	0VS3	0VS80
Cre16.g663100	ALG7	PF00953	2.46	2.19	5.21	−0.12	0.47
Cre13.g585850	ALG13	PF04101	1.25	1.07	3.60	−0.21	0.89
Cre16.g667701	ALG14	PF08660	3.28	2.60	4.65	/	Inf up
Cre12.g516550	ALG1	PF00534	2.99	1.82	3.27	−0.64	−0.49
Cre02.g095147	ALG2	PF00534	–	–	–	–	–
Cre01.g058521	ALG11	PF00534	–	–	–	–	–
Cre01.g041300	ALG10	PF04922	2.66	2.87	3.23	0.18	−0.35
Cre16.g690150	ALG6	PF03155	–	–	–	–	–
Cre09.g414250	ALG8	PF03155	–	–	–	–	–
Cre16.g652850	ALG5	PF00535	–	–	–	–	–
Cre12.g523300	RPN1	PF04597	6.03	3.91	2.16	−0.53	−2.10
Cre08.g368450	RPN2	PF05817	0.82	1.14	4.74	0.52	1.73
Cre07.g330100	STT3A	PF02516	–	–	–	–	–
Cre09.g387245	STT3B	PF02516	0.02	0.19	0.74	/	4.60
Cre16.g675602	OST3/6	PF04756	0.43	0.57	1.37	–	1.07
Cre02.g097150	OST5	PF05251	3.32	4.47	3.26	0.38	−0.70

*N-glycosylation pathway was proposed based on the genome of C. reinhardtii v5.5 in silico. ALG7, N-acetylglucosamine phosphostransferase; ALG13, Beta-1,4-N-acetylglucosaminyl transferase; ALG14, Beta-1,4-N-acetylglucosaminyl transferase; ALG1, Beta-1,4-mannosyl transferase; ALG2, Alpha-1,3-mannosyltransferase; ALG11, Alpha-1,2-mannosyltransferase; RFT, Flippase; ALG10, alpha-1,2 glucosyltransferase; ALG6, alpha-1,3-glucosyltransferase; ALG8, alpha-1,3-glucosyltransferase; ALG5, Dolichol-phosphate glucosyltransferase; RPN1, ribophorin I; RPN2, ribophorin II; STT3A, DDPGT subunit a; STT3B, DDPGT subunit b; OST3/6, OST complex subunit 3/6; OST5, OST complex subunit 5. “–” means the expression was not detected. “/” means the DESeq_log2FC was not counted.*

**FIGURE 2:**
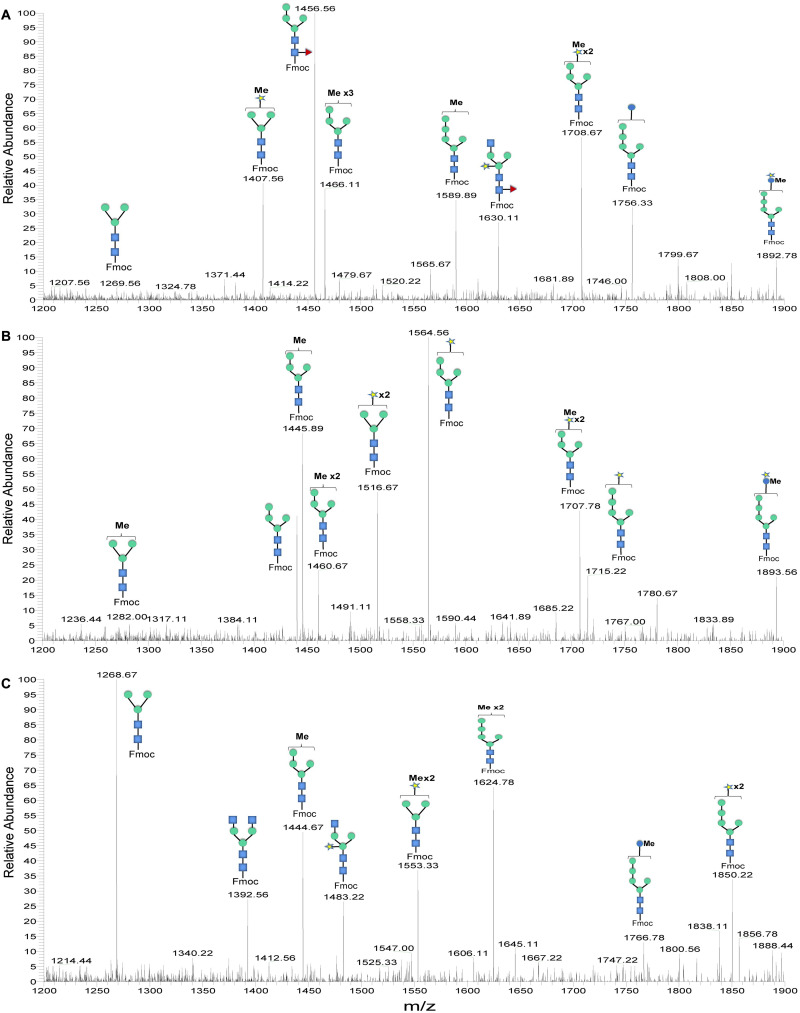
ESI mass spectra of Fmoc-labeled N-glycans released from *Chlamydomonas reinhardtii* soluble proteins using PNGase F. **(A)** Control group; **(B)** low lead stress; **(C)** high lead stress. Me, methylation; blue square, N-acetylglucosamine; green circle, mannose; white star, xylose; red triangle, fucose; blue circle, glucose; ×2, twice. Monosaccharides and Me depicted above the solid horizontal bracket can be bound to any subjacent residue. Consortium of Functional Glycomics nomenclature was used for the N-glycan structures (Varki et al., 2009).

Although bioinformatic analyses gave rise to thorough insights into the N-glycosylation pathway in the ER of *C. reinhardtii* and the expression of related genes from transcriptome analysis, no information about N-glycans of glycoproteins that happened under the lead stress can be inferred. To investigate the impact of lead stress on the biosynthesis of glycans N-linked to proteins, N-glycans from control, low lead and high lead stressed cells were released from total intracellular protein by PNGase F. The released N-glycans were then labeled with Fmoc-Cl and analyzed by ESI-MS. The results are presented in Figure 2. Based on the m/z values of [M+Na]^+^ ions, ions were majorly assigned to Fmoc derivatives of structure from Man_3_GlcNAc_2_ to Man_5_GlcNAc_2_. Nevertheless, no obvious ion was assigned to high mannose structures, such as Man_8–9_GlcNAc_2_. Furthermore, a linear Man_5_GlcNAc_2_ oligosaccharide was observed from three groups.

N-glycan profiles were different among the three groups. To characterize the difference of N-glycan profiles under the third day of the lead stress, the relative abundance of N-glycan structures was compared, as shown in Table 2. Compared with N-linked glycans in the control group, fucose modifying N-glycans (i.e., GlcNAc_1_Man_3_Xyl_1_Fuc_1_GlcNAc_2_ and Man_4_Fuc_1_GlcNAc_2_) were not observed under the lead stress. In addition to these two fucose modifying N-glycans, some N-glycan structures were also not identified from the total glycoproteins under the lead stress, including Glc_1_Man_5_GlcNAc_2_, Me_3_Man_4_GlcNAc_2_, Me_1_Man_5_GlcNAc_2_, and Xyl_1_Me_1_Man_3_GlcNAc_2_. Differently, Me_1_Man_4_GlcNAc_2_ was the unique oligosaccharide structure identified under the low and high lead stresses but not in the control group. Diversely, complex oligosaccharide Me_2_Man_5_GlcNAc_2_, GlcNAc_2_Man_3_GlcNAc_2_, GlcNAc_1_Man_3_Xyl_1_GlcNAc_2_, Glc_1_Me_1_Man_5_GlcNAc_2_, Xyl_2_Man_5_ GlcNAc_2_, and Xyl_1_Me_2_Man_3_GlcNAc_2_ structures were identified under the high lead stress but not under the control and low lead stress. Also, it was shown that the relative abundance of Me_2_Man_5_GlcNAc_2_ oligosaccharide structure was a little bit higher than that of the other oligosaccharide structures. Furthermore, converse to oligosaccharides Xyl_2_Me_1_Man_4_GlcNAc_2_ and Glc_1_Xyl_1_Me_1_Man_5_GlcNAc_2_ in control and low lead stress groups, these two complex N-glycans were not remarkably observed in the high lead stress group. It was observed that oligosaccharides Man_4_Fuc_1_GlcNAc_2_ (28.64%), Xyl_1_Man_4_GlcNAc_2_ (26.83%), and Man_3_GlcNAc_2_ (28.45%) had the highest relative abundance under the control group, low lead stress, and high lead stress, respectively.

**TABLE 2:** Relative abundance (RA) of the N-glycans found on *Chlamydomonas reinhardtii* soluble proteins under the third day of lead stress.

N-glycan Structures	Nomenclature	RA in control group	RA in low Pb stress	RA in high Pb stress
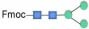	Man_3_GlcNAc_2_	1.2%	trace	28.45%
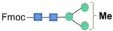	Me_1_Man_3_GlcNAc_2_	Trace	1.46%	Trace
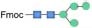	Man_4_GlcNAc_2_	Trace	11.06%	Trace
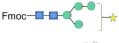	Xyl_1_Man_4_GlcNAc_2_	Trace	26.83%	Trace
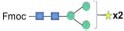	Xyl_2_Man_3_GlcNAc_2_	Trace	13.15%	Trace
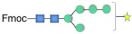	Xyl_1_Man_5_GlcNAc_2_	Trace	5.75%	Trace
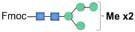	Me_2_Man_4_GlcNAc_2_	Trace	6.20%	Trace
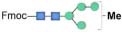	Me_1_Man_4_GlcNAc_2_	Trace	18.33%	14.08%
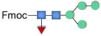	Man_4_Fuc_1_GlcNAc_2_	28.64%	Trace	Trace
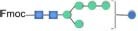	Glc_1_Man_5_GlcNAc_2_	9.25%	Trace	Trace
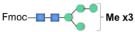	Me_3_Man_4_GlcNAc_2_	11.11%	Trace	Trace
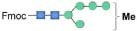	Me_1_Man_5_GlcNAc_2_	10.01%	Trace	Trace
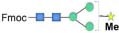	Xyl_1_Me_1_Man_3_GlcNAc_2_	11.68%	Trace	Trace
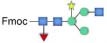	GlcNAc_1_Man_3_Xyl_1_Fuc_1_GlcNAc_2_	7.77%	Trace	Trace
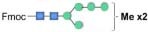	Me_2_Man_5_GlcNAc_2_	Trace	Trace	18.32%
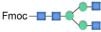	GlcNAc_2_Man_3_GlcNAc_2_	Trace	Trace	7.75%
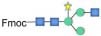	GlcNAc_1_Man_3_Xyl_1_GlcNAc_2_	Trace	Trace	7.54%
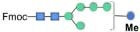	Glc_1_Me_1_Man_5_GlcNAc_2_	Trace	Trace	3.70%
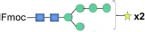	Xyl_2_Man_5_GlcNAc_2_	Trace	Trace	9.63%
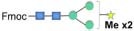	Xyl_1_Me_2_Man_3_GlcNAc_2_	Trace	Trace	10.49%
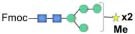	Xyl_2_Me_1_Man_4_GlcNAc_2_	16.31%	11.51%	Trace
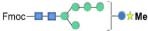	Glc_1_Xyl_1_Me_1_Man_5_GlcNAc_2_	3.99%	5.66%	Trace

Additionally, genes involved in the core region of the ERQC (CNX, CRT, GI, GIIA, GIIB, UGGT, and PDIs) systems were also analyzed from the transcriptome of *C. reinhardtii* under the lead stress. It was shown that only PDIs were differentially expressed and significantly upregulated under the third day of high lead stress (Table 3). In the genome of *C. reinhardtii*, eight PDI genes were annotated to PDI1-5, PDIA1, and PDIA6. Although the eighth PDI was only predicted to have a thioredoxin domain, therefore, it was speculated to be PDIx here. Among all these eight PDI genes, PDI2, PDIA1, PDIA6, and PDIx were upregulated under the high lead stress.

**TABLE 3:** Expression of genes involved in the core region of ERQC system in *Chlamydomonas reinhardtii* under the third day of lead stress.

Gene ID	Abbreviation	Domain	FPKM			DESeq_log2FC	
			0	3	80	0VS3	0VS80
Cre07.g357900	CNX	PF00262	0.39	0.31	1.13	−0.23	0.34
Cre01.g038400	CRT	PF00262	–	–	–	–	–
Cre13.g579734	GI	PF03200	1.02	0.87	1.19	−0.21	−0.42
Cre03.g190500	GIIA	PF01055	–	–	–	–	–
Cre17.g725350	GIIB	PF12999	3.76	3.58	11.37	0.03	1.01
Cre05.g233303	UGGT	PF06427	0.65	1.40	1.65	0.97	1.07
Cre12.g518200	PDI1	PF00085	3.49	3.58	7.34	0.11	0.45
Cre01.g033550	PDI2	PF00085	3.78	4.92	22.08	0.40	1.83
Cre16.g692751	PDI3	PF00085	17.45	16.39	30.76	−0.02	0.19
Cre07.g328150	PDI4	PF00085	8.69	8.16	20.77	−0.14	0.40
Cre02.g104350	PDI5	PF00085	1.37	1.01	3.92	−0.37	0.89
Cre02.g088200	PDIA1	PF00085	12.01	9.03	84.60	−0.40	2.54
Cre07.g326600	PDIA6	PF00085	0.61	0.40	2.72	−0.56	1.51
Cre09.g391900	PDIx	PF00085	11.76	11.28	64.78	0.01	1.84

*CNX, calnexin; CRT, calreticulin; GI, glucosidase I; GIIA, glucosidase II, alpha-subunit; GIIB, glucosidase II, beta-subunit; UGGT, UDP-glucose, glycoprotein glucosyltransferase; PDI, protein disulfide isomerase.*

**FIGURE 3:**
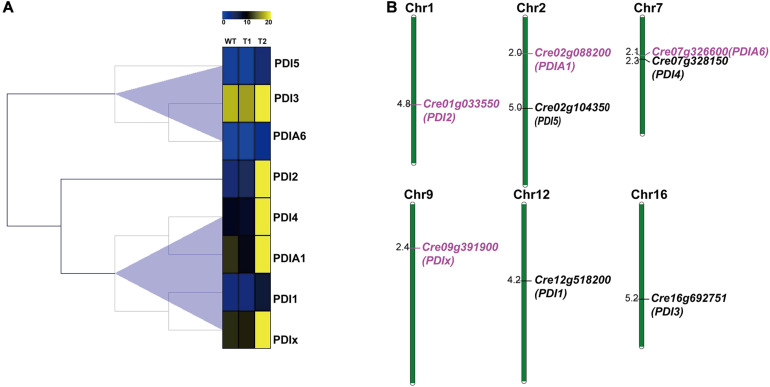
Expression profiles and chromosome distributions of eight PDI genes in *Chlamydomonas reinhardtii*. **(A)** Hierarchical clustering of expression of eight PDI genes in *C. reinhardtii*. **(B)** Eight PDI genes are distributed to six chromosomes. Number of chromosomes was presented at the top of each chromosome. Some other chromosomes without PDI gene location were not shown in this figure. Scale on the left of each chromosome in megabases (Mb). Genes in purple represent the upregulation expression under the high lead stress.

To further study the PDI genes, bioinformatic analysis was carried out. The expression profiles of eight PDIs were shown in Figure 3A. Obviously, PDI2, PDIA1, PDIA6, and PDIx were all upregulated. In the genome of *C. reinhardtii*, 17 nuclear chromosomes were found. The positions of the identified PDI genes were drafted to chromosomes by using Mapchart software. Ultimately, eight PDI genes were located on six chromosomes (Figure 3B). Obviously, there is only one PDI gene on each of 1, 9, 12, and 16 chromosomes. They were PDI2 (Cre01g033550), PDIx (Cre09g391900), PDI1 (Cre12g518200), and PDI3 (Cre16g692751), respectively. Additionally, two PDI genes were distributed on chromosomes 2 and 7. They were PDIA1 (Cre02g088200) and PDI5 (Cre02g104350) on chromosome 2 and PDIA6 (Cre07g326600) and PDI4 (Cre07g328150) on chromosome 7. Contrary to PDIA1 and PDI5 genes anchored remotely on chromosome 2, whereas PDIA6 and PDI4 genes were clustered within a very short distance on chromosome 7, less than 0.2 Mb. It was also shown that the four upregulated genes (PDI2, PDIA1, PDIA6, and PDIx) were located on four different chromosomes.

**FIGURE 4:**
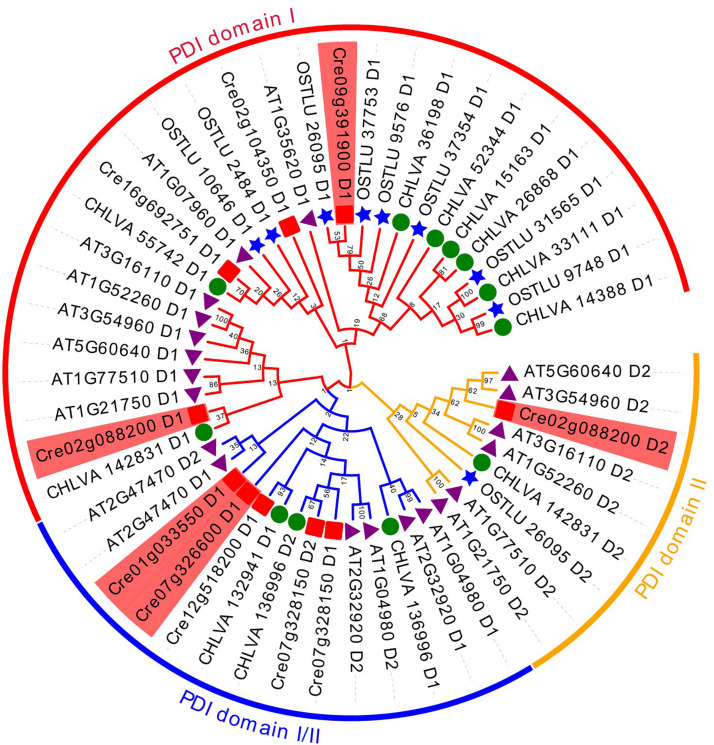
Unrooted phylogenetic analysis of PDI genes from different green algae and plants using the domain sequences. Neighbor-joining (NJ) tree with 1,000 bootstrap replicates was reconstructed using MEGA 6 software and online EvolView. Four green algal species are *Chlamydomonas reinhardtii*, *Ostreococcus lucimarinus*, *Chlorella variabilis*, and plant *Arabidopsis thaliana*. Different colored arcs/branches are different groups of PDI domains, including red arc for PDI domain I, orange arc for PDI domain II and blue arc for PDI domain I/II. Solid red rectangles represent PDI domains from *C. reinhardtii*. Solid blue stars represent PDI domains from *O. lucimarinus*. Solid green circles represent PDI domains from *C. variabilis*. Solid purple triangles represent PDI domains from plant *A. thaliana*. Five domains in red background are from four upregulated PDI genes in *C. reinhardtii*.

Fourteen PDI proteins were obtained from *A. thaliana* by using HMM and BLASTP. As annotated in the previous article, all these 14 PDIs were named AtPDI1-14 in *A. thaliana* (Yuen et al., 2016). Meanwhile, 8 and 10 PDI proteins were, respectively, achieved from the genomes of green algae *O. lucimarinus* and *C. variabilis*. All PDIs harbor one or more catalytic domains; the catalytic domains are homologous to the redox protein, thioredoxin. Therefore, the protein sequences of catalytic domains were obtained from all these PDI proteins and phylogenetically analyzed (Figure 4). Based on phylogenetic results, all catalytic domains were classified into three groups, namely, PDI domain I, PDI domain II, and PDI domain I/II groups. The PDI domain I group was the largest group and contained 28 catalytic domains. The PDI domain II group included nine PDI catalytic domains. Specifically, some PDI domains I and II were mixed in the third group, called PDI domain I/II group. It contained 14 domains, nine domain I and five domain II. Among four upregulated genes, PDIA1 (Cre02g088200) was predicted to have two thioredoxin domains; one domain was clustered into the PDI domain I group, and the other one was assembled with the PDI II domain group. In contrast, the other three PDIs only harbored one thioredoxin domain. As shown in Figure 4, the catalytic domain of PDIx (Cre09g391900) was grouped in PDI domain I. The catalytic domains of PDI2 (Cre01g033550) and PDIA6 (Cre07g326600) were clustered together within the PDI domain I/II group.

**FIGURE 5:**
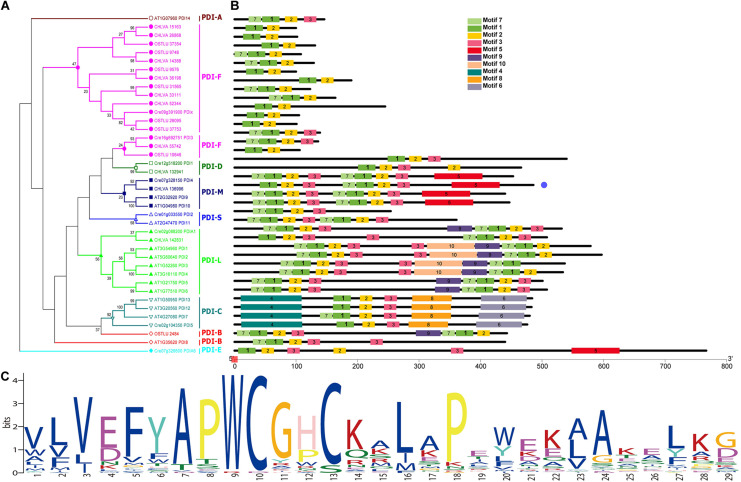
Phylogenetic relationships and conserved motifs of PDI genes from algae and plant. **(A)** Phylogenetic tree was constructed based on the full-length sequences of algal and plant PDI proteins using MEGA 6 software. Protein in dark red belongs to PDI-A subfamily (hollow circle). Proteins in purple belong to PDI-F subfamily (solid circle). Proteins in dark green belong to PDI-D subfamily (hollow box). Proteins in dark blue belong to PDI-M subfamily (solid box). Proteins in light blue belong to PDI-S subfamily (hollow triangle). Proteins in green belong to PDI-L subfamily (solid triangle). Proteins in cyan belong to PDI-C subfamily (inverted hollow triangle). Proteins in red belong to PDI-B subfamily (hollow rhombus). Proteins in aqua green belong to PDI-E subfamily (solid rhombus). **(B)** Motif structure of all PDI proteins. Motifs, numbers 1–10, are shown in different colored boxes. Detailed sequence information for each motif is presented in Supplementary Figure 1. Length of each PDI protein can be estimated using the scale at the bottom of the structure of conserved motifs. **(C)** The sequence logo for motif I (thioredoxin domain).

Phylogenetic analysis of PDI full-length sequences and their predicted motifs are illustrated in Figure 5. Based on the phylogenetic and motif analysis, all these PDI proteins were classified into night subfamilies, including PDI-A, PDI-B, PDI-C, PDI-D, PDI-E, PDI-F, PDI-M, PDI-S, and PDI-L subfamilies (Figures 5A,B). A search on the MEME program identified 10 motifs in all PDI proteins. The number of motifs in PDIs ranged from 2 to 10, and the length of motifs varied from 21 (motifs 2–3 and motif 7) to 100 (motif 4) amino acids (Figure 5B). All PDIs from the three green algae (*C. reinhardtii*, *O. lucimarinus*, and *C. variabilis*) were not clustered with PDI-A protein from terrestrial plant *A. thaliana*. The next two purple branches were named PDI-F with a motif 1 (a thioredoxin domain) and a C-terminal alpha helix. These PDI-F proteins were very short, ranging from 131 to 245 amino acids. Two PDI proteins, respectively, from *C. reinhardtii* (Cre12g518200_PDI1) and *C. variabilis* (CHLVA_132941) were composed of 540 and 466 amino acids. These two proteins displayed a central thioredoxin domain with a standard YAPWCGHC active sequence (Figures 5A–C). These two specific proteins were grouped into the PDI-D subfamily. Four proteins (Cre07g328150_PDI4, CHLVA_136996, AT2G32920_PDI9, and AT1G04980_PDI10) were divided into PDI-M subfamily. All these four PDI-M proteins possessed two thioredoxin domains with a strictly conserved WCGHC active site. A slightly modified ER retention sequences were observed at the C-terminus of proteins, [K/V/N] [E/D/G] [E/D] L. Considering the absence of motifs 9 and 10 in PDI-M compared with PDI-L, these four proteins (440–486 amino acids) were slightly shorter than that found in PDI-L (501–597 amino acids).

In this phylogenetic analysis, only two proteins (Cre01g033550_PDI2 and AT2G47470_PDI11) were grouped into the PDI-S subfamily, as shown in Figure 5A. It was observed that PDI11 from *A. thaliana* contained two thioredoxin domains, whereas *Chlamydomonas* PDI2 possessed only one thioredoxin domain. The prediction of protein secondary structure showed that these two PDI-S proteins contained a putative alpha-helical conformation and a predicted peptide/protein binding position on the C-terminus. Eight proteins belonging to the PDI-L subfamily were clustered into one branch. These PDI-L proteins were predicted to have two thioredoxin domains (motif I). It was shown that all the PDI-L proteins contained a classical C-terminal ER retention signal (KDEL). In addition to motif 1, all PDI-L proteins harbored conserved motif 2, 3, and 7. Some PDI-L proteins were predicted to have additional motifs, such as motif 9 and 10 in the PDI-L subfamily. In *C. reinhardtii*, PDIA1 was phylogenetically clustered into this PDI-L subfamily.

The next subfamily is PDI-C in a cyan mark. All these PDI-C proteins contained one thioredoxin domain and some other conserved motifs, including motifs 2, 3, 4, 6, and 8. Motif 4 was predicted to be the N-terminal of ER-Golgi intermediate compartment clusters from the pfam analysis. In contrast, motifs 6 and 8 were COPII coated ER vesicle transporter domains. Additionally, the search of protein sequences showed that all PDI-C proteins without N-terminal signal peptide contained two predicted transmembrane anchoring domains on the terminus. PDI5 harboring these characteristics was classified into this PDI-C subfamily in *C. reinhardtii*. PDIA6 from *C. reinhardtii* formed an individual clade in this phylogenetic tree. It was the largest PDI protein with 767 amino acids. Analysis of signal peptide, a transmembrane domain, and conserved PDI domain showed that PDIA6 included one putative thioredoxin domain and a predicted TMD. Compared with PDI-E in *Arabidopsis* (Selles et al., 2011), PDIA6 was primarily classified into the PDI-E subfamily in this study.

### Endoplasmic Reticulum-Associated Degradation Pathway in *C. reinhardtii* Under Lead Stresses

Incomplete/misfolded glycoproteins will be removed from the ERQC system after multiple CNX/CRT cycles and degraded by an ERAD pathway. What happened in the ERAD pathway of *C. reinhardtii* under the lead stress? A total of 43 genes with detected expression were identified from the transcriptome of *C. reinhardtii* under the third day of lead stress (Table 4). All 43 ERAD-related genes were not differentially expressed under the low lead stress. However, 14 genes were significantly upregulated under high lead stress. These upregulated genes were subdivided into different groups, including Uba, Ubc, Ube, Ubx, Ufd, Ubp, and Otu groups.

**TABLE 4:** Expression of putative genes involved in the ERAD pathway in *Chlamydomonas reinhardtii* under the third day of lead stress.

Gene ID	Abbreviation	Domain	FPKM			DESeq_log2FC	
			0	3	80	0VS3	0VS80
Cre06.g257900	OS9	PF07915	1.13	0.60	3.33	−0.70	0.99
Cre03.g197400	Hrd3A-1	PF08238	2.25	1.90	3.86	−0.15	0.12
Cre01.g026300	Hrd3A-2	PF08238	1.98	1.87	3.19	0.12	0.63
Cre15.g636400	Der1-1	PF04511	16.94	15.44	16.67	−0.09	−0.67
Cre12.g523500	Der1-2	PF04511	31.33	25.13	47.39	−0.26	−0.10
Cre06.g296983	Uba2	PF10585	0.20	0.14	1.89	−	2.58
Cre12.g546650	Ubc7	PF00179	157.11	132.57	98.65	−0.18	−1.30
Cre01.g026600	Ubc13	PF00179	57.45	35.16	57.38	−0.62	−0.57
Cre06.g292800	Ubc4/5	PF00179	9.66	8.50	37.95	−0.10	1.36
Cre12.g515450	Ubc2	PF00179	45.09	36.10	47.81	−0.27	−0.54
Cre01.g046850	Ubc1	PF00179	8.09	8.93	9.68	0.19	−0.38
Cre13.g570300	Ubc24	PF00179	0.08	0.04	0.85	−	2.66
Cre05.g240150	Ubc16	PF00179	7.46	5.20	9.49	−0.46	−0.28
Cre08.g370850	Ubc12	PF00179	91.36	92.17	99.47	0.08	−0.51
Cre01.g027200	Ubc8	PF00179	151.14	158.93	188.97	0.14	−0.30
Cre01.g019450	Ubc9	PF00179	0.33	0.34	0.51	−	−
Cre02.g141450	Ubc14	PF00179	22.61	21.27	25.65	0.04	−0.41
Cre06.g297650	UbcQ	PF00179	0.15	0.23	2.08	0.55	2.45
Cre12.g510300	Ubc21	PF00179	0.11	0.14	2.14	0.36	3.62
Cre03.g202897	UbcJ1	PF00179	25.25	24.60	31.58	0.04	−0.31
Cre08.g372400	UbcJ2	PF00179	15.86	16.91	18.77	0.16	−0.38
Cre01.g012450	Ube3-6	PF00632	0.90	1.04	4.45	0.25	1.68
Cre06.g280300	Ube3-1	PF00632	10.14	9.09	18.72	−0.09	0.26
Cre08.g364550	Ube3A-1	PF00632	3.21	4.41	16.88	0.46	1.28
Cre02.g099100	Ube3-8	PF00632	0.27	0.44	1.59	0.78	1.94
Cre11.g467609	Ube3-3	PF00632	18.67	18.23	16.94	−0.03	−0.24
Cre07.g312900	Ube3-4	PF00632	14.72	16.14	20.76	0.28	−0.03
Cre10.g433900	Ube3-5	PF00632	7.87	9.36	11.12	0.10	−0.05
Cre12.g533750	Ube3-7	PF00632	0.67	0.63	3.05	−0.07	1.55
Cre01.g022100	Ube3-2	PF00632	7.06	7.51	14.82	0.16	0.45
Cre02.g099100	Ube3A-2	PF00632	0.27	0.44	1.59	0.78	1.94
Cre12.g517850	Doa1	PF12906	0.61	0.59	1.57	0.03	0.74
Cre09.g400738	Doa2	PF12906	24.41	23.41	32.74	0.63	0.10
Cre17.g747747	Doa3	PF12906	8.03	10.87	9.03	0.53	−0.42
Cre01.g034451	Ubx1-1	PF00789	4.70	4.21	25.35	−0.10	1.81
Cre01.g030550	Ubx1-2	PF00789	1.49	1.13	6.62	−0.33	1.52
Cre06.g293051	Npl4	PF05021	8.59	10.13	25.70	0.32	0.95
Cre14.g613350	Ufd1-1	PF03152	6.48	6.93	15.01	0.16	0.64
Cre03.g179100	Ufd1-2	PF03152	9.23	11.02	41.65	0.28	1.52
Cre09.g398350	Ufd2	PF10408	0.07	0.14	2.68	−	4.55
Cre08.g366400	Rad23	PF00627	21.05	19.76	75.78	−0.03	1.21
Cre02.g080350	Ubp14	PF00443	1.08	0.95	6.47	−0.16	1.94
Cre09.g409050	Otu1	PF02338	6.40	5.72	23.29	−1.31	3.11

*OS, putative osteosarcoma; Hrd, HMG-CoA reductase degradation protein; Der, Derlin; Uba, ubiquitin-activating enzyme E1; Ubc, ubiquitin-conjugating enzyme E2; Ube, ubiquitin-protein ligase E3; Doa, putative degradation of alpha-2 protein; Ubx, ubiquitin regulatory X domain-containing protein; Npl, substrate-recruiting factor Npl; Ufd, ubiquitin fusion degradation protein; Rad, ubiquitin receptor Rad23; Ubp, ubiquitin-specific protease; Otu, ubiquitin thioesterase Otu1. “–”: the expression of genes was extremely low in RNAseq. Negative fold changes represent downregulation, whereas positive fold changes represent upregulation under lead stresses. All presented fold changes are statistically significant, q-value < 0.05.*

## Discussion

In algae, Pb can change the algal ultrastructure, effect the growth, photosynthesis, respiration, and the activity of related enzymes, and reduce polysaccharides and protein concentrations. In agreement with previous studies in *Chlorella sorokiniana* 211-8k, *Chlorella* sp. FleB1, and *Scenedesmus* YaA6, the growth of *C. reinhardtii* was also inhibited under the high lead stress here (Carfagna et al., 2013; Dao and Beardall, 2016). Meanwhile, the concentration of soluble protein was significantly reduced in *C. reinhardtii* under the high lead stress; this result was consistent with a decrease of soluble protein levels in green microalga *C. sorokiniana* (Carfagna et al., 2013). Additionally, many studies showed that protein levels could be affected by lead stress (Harrison et al., 1997; Shanab et al., 2012; Qiao et al., 2013; Dao and Beardall, 2016; Li et al., 2016). However, it is still unclear why the protein levels were changed under lead stress.

An efficiently and accurately ERQC system was important in the protein quality control and protein level, as it could differentiate terminally incomplete/misfolded proteins, stopped the futile CNX/CRT folding cycles of the proteins, and eliminated them via a multistep degradation process called ERAD (Smith et al., 2011). It was already known that more than half of secretory and membrane proteins were co-translationally N-glycosylated when transporting into ER (Lizak et al., 2011). Correctly N-linked structures of proteins played an important role in protein folding and quality control (Aebi et al., 2010). *In silico* analysis of the *C. reinhardtii* genome, ALG7, ALG13/14, ALG1, ALG2, ALG11, ALG5, ALG6, ALG8, and ALG10 were identified. Among them, ALG13/ALG14 catalyzes the addition of the second core GlcNAc residue to Dol-PP-GlcNAc (Lannoo and Van Damme, 2015). In this study, the ALG14 gene was a unique differentially expressed ALG gene under the high lead stress from the transcriptome analysis of *C. reinhardtii*. Putative *C. reinhardtii* ALG14 exhibited 40% of identity with ALG14 from *A. thaliana* (Mathieu-rivet et al., 2013). It was certified that ALG14 organized the formation of a multiglycosyltransferase complex during the initiation of N-glycan biosynthesis (Kellokumpu et al., 2016). Oligosaccharyltransferase (OST), gatekeeper to the secretory pathway, catalyzes the first step in the biosynthesis of N-glycoproteins (Dempski and Imperiali, 2002). Six subunits of the OST complex (Ribophorin I and II, STT3A, STT3B, OST3/6, and OST5) were predicted from the *C. reinhardtii* genome. STT3A and STT3B are catalytically active subunits of heteromeric membrane-bound protein OST complex. Ribophorin I was downregulated, and Ribophorin II and STT3B were upregulated in *C. reinhardtii* under the high lead stress. It was displayed that putative *C. reinhardtii* STT3A/STT3B exhibited 27% (At5g19960) and 57% (At1g34130) of identity with orthologs from *A. thaliana* (Mathieu-rivet et al., 2013). On the contrary to the STT3B-deficiency, STT3A-deficient plants displayed an obvious change in N-glycosylation efficiency and affected the biogenesis of heavily glycosylated proteins (e.g., the pattern recognition receptor) (Nekrasov et al., 2009; Häweker et al., 2010). However, *stt3a* and *stt3b* double knockout were gametophytic lethal in *A. thaliana*, suggesting that these two catalytic subunits are very important for the N-glycosylation of plant proteins (Koiwa, 2003). Therefore, it was speculated that the upregulated STT3B was important in the N-glycosylation of *Chlamydomonas* proteins under the lead stress.

Owing to the four differentially expressed genes (ALG14, RPN1, RPN2, and STT3B genes) in *C. reinhardtii*, N-glycan structures were also analyzed in this study. So far, N-glycosylation of proteins in *C. reinhardtii* has received very little attention. The first study in 2011 showed that *C. reinhardtii* harbored glycoproteins with mammalian-like sialylated N-linked glycans (Mamedov and Yusibov, 2011). Glycoproteins from *C. reinhardtii* were also found to carry oligomannosidic N-glycans (Man_2–5_GlcNAc_2_) containing 6-O-methylated mannoses and one or two xylose residues (Mathieu-rivet et al., 2013). Subsequently, branched Man_5_GlcNAc_2_ N-glycans were re-evaluated as being linear oligosaccharide chains based on the analysis of ESI-MS^n^ (Vanier et al., 2017). Compared with these studies in *C. reinhardtii*, consistent N-glycan structures with Man5 were also identified in this study via the analysis of ESI-MS under the lead stress. Compared with control and low lead stress, the relative abundance of Man_3_GlcNAc_2_ was extremely high under the high lead stress. A similar result was reported by a previous study, where it showed that Man_3_GlcNAc_2_ glycan was accumulated, protein N-glycosylation was reduced, and the UPR was activated by osmotic stress and hormone treatment in ALG11 mutant, indicating that protein N-glycosylation was important for plant development and the response to abiotic stresses (Zhang et al., 2009). Six specific N-glycans were only observed under high lead stress, although the two N-glycans from glycoproteins were found under the control and low lead stress but not under the high lead stress. One possible explanation for the present or absent N-glycans under the high lead stress is that these modifications were important for the specific functions of glycoproteins to alleviate the Pb stress in the alga. Another possible explanation is that these specific N-glycosylated proteins were accumulated in the ER, stimulating a UPR and finally degraded via the ERAD pathway. The complex N-linked glycans were widely studied in plant stress tolerance, such as Man_4_GlcNAc_2_ and Man_5_GlcNAc_2_ (Frank et al., 2008; von Schaewen et al., 2008; Nagashima et al., 2018). To our knowledge, the effects of altered N-glycan structures in microalga under the abiotic stress was the first time to be reported here.

Interestingly, in the core region of ERQC, PDI genes were only significantly upregulated under the lead stress. PDI, a molecular chaperone, is involved in the formation, reduction, and isomerization of disulfide bonds for the correct folding of nascent proteins in ER and prevention of misfolding during stress (Strasser, 2020). PDI could be recruited by CNX/CRT for the high-specificity binding between GlcMan9GlcNAc2 and CNX/CRT during the ERQC system (Liu and Li, 2014). The *Arabidopsis* genome encodes 14 PDI-like proteins harboring one or two thioredoxin-like domains; they are classified into six PDI subfamilies (PDI-A, PDI-B, PDI-C, PDI-L, PDI-M, and PDI-S) (Strasser, 2020). Eight PDI-like proteins (CrPDI1-5, CrPDIA1, CrPDIA6, and CrPDIx) were annotated in *C. reinhardtii* genome. Among the eight PDI-like proteins, four upregulated PDI genes (CrPDI2, CrPDIA1, CrPDIA6, and CrPDIx) were found from the transcriptome of *C. reinhardtii* under high lead stress. In a previous article, CrPDI2 was certified to regulate circadian rhythm and interact with peroxiredoxin, especially during night in *C. reinhardtii* (Filonova et al., 2013). Although CrPDI2 contained one thioredoxin domain, it was clustered with AtPDI11 harboring two thioredoxin domains in the PDI-S subfamily of *Arabidopsis*. The function of AtPDI11 has remained unknown so far (Yuen et al., 2016). The thioredoxin domain of CrPDI2 (Cre02g033550 D1) and domains from AtPDI11 (AT2G47470 D1/D2) were together clustered into a monophyletic group in PDI domain I/II, indicating that the appearance of a second domain in *A. thaliana* might result from duplication events during chlorophyte evolution. A similar relationship was also observed in terrestrial plants, and it was supported by the fact that genome duplication and rearrangements were important in the evolution of plants (Selles et al., 2011). CrPDIA1 harboring two thioredoxin domains and an ER retention domain KEDL was divided into the PDI-L subfamily with six PDI from *A. thaliana*. The mechanism of ERQC was perturbed in the AtUGGT mutant and finally caused the upregulation of the PDI-L2-1 gene (Blanco-Herrera et al., 2015). However, CrPDIA6 containing one thioredoxin domain and long protein sequences was clustered in an independent clade PDI-E. The specific structure suggested that CrPDIA6 might evolve independently from other PDIs. CrPDIx formed a large clade with PDI-F subfamily members. It was known that PDI could aid the correct folding and assembly of nascent proteins in ER to prevent the aggregation of proteins under some unfavorable environmental conditions, such as heat and drought stresses (Branlard et al., 2020). The role of increased PDI protein was to enhance the salt tolerance of *Medicago sativa* L (Rahman et al., 2015). Therefore, it is reasonable to speculate that the upregulation of four CrPDI genes was important for the tolerance of *C. reinhardtii* under the lead stress. Together with the upregulation of CrPDI genes in this study, PDI proteins were also differentially expressed in response to other metal stresses, such as arsenic (As), cadmium (Cd), and copper (Cu). The exposure of *A. thaliana* to Cd showed the upregulation of PDI protein (Sarry et al., 2006). The expression of PDI protein was reduced by excessive As- or Cu-treatments in rice *Oryza sativa* (Ahsan et al., 2007a, 2008). However, the PDI content was increased by exposure to excessive Cd (Ahsan et al., 2007b). It was suggested that the expression of PDI protein was dependent on species and metals. Furthermore, a previous study indicated that PDI played a vital role in the first committed step of the ERAD pathway under metal stresses (Anelli et al., 2007; Liu et al., 2016). PDIs in plants and animals were verified to form a complex with incomplete/misfolded proteins for the following degradation under the stresses (Liu and Li, 2014; Strasser, 2020), whereas the mechanism of algal PDIs in retaining incomplete/misfolded proteins is still unknown.

**FIGURE 6:**
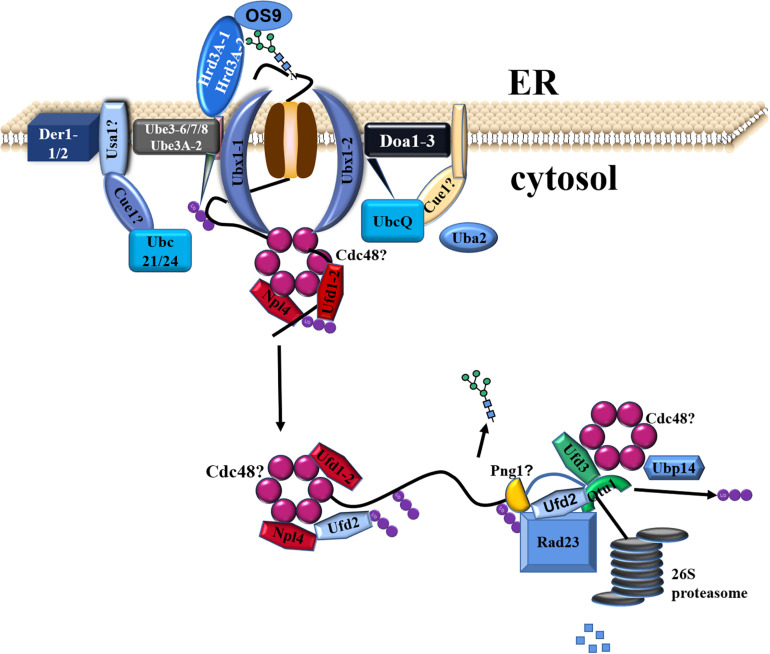
Proposed ERAD model in *Chlamydomonas reinhardtii* under the third day of lead stress.

Incomplete/misfolded proteins from the CNX/CRT cycle were finally eliminated in the ERAD pathway through step-wise procedures (Smith et al., 2011). Incomplete/misfolded proteins were increasingly accumulated to cause ER stress under biotic and/or abiotic stresses. Subsequently, UPR was activated to alleviate ER stress via different mechanisms, e.g., inhibition of protein synthesis, suppression of entry of other proteins into the ER, enhancement of protein folding, and degradation (Grootjans et al., 2016; Vincenz-Donnelly and Hipp, 2017). In this study, genes involved in ER stress and UPR were not found to be differentially expressed in *C. reinhardtii* under the lead stress. However, genes related to ERAD (e.g., Uba, Ubc, Ube, Ubx, Ufd, Ubp, and Otu) were significantly upregulated, and a putative working model was proposed, as shown in Figure 6. The first step is the recruitment of ERAD substrates. The *C. reinhardtii* genome has three OS9/Hrd3 homologous, CrOS9, CrHrd3A-1, and CrHrd3A-2 (Figure 6). Hrd3 could recognize and bind hydrophobic amino acid residues of misfolded proteins and make the initial selection of a potential ERAD substrate (Hirsch et al., 2009). OS9 was certificated to specifically recognize and bind N-glycans with alpha-1,6-Man residue (Hosokawa et al., 2010). After these recognitions, only terminally incomplete/misfolded proteins were recruited for degradation. However, these three genes were not differentially expressed in this study. The second step is the retrotranslocation of ERAD substrates from the ER lumen to the cytosolic surface of the ER membrane for their ubiquitination (Figure 6). This step is almost unknown in plants and algae. The third step is the ubiquitination of chosen ERAD substrates. The typical ubiquitination reaction was performed by a three-step process, including the activation by a ubiquitin-activating enzyme (E1/Uba), conjugation by the ubiquitin-conjugating enzyme (E2/Ubc), and ligation catalyzed by E3 ligase (Ube) (Pickart, 2004). In this study, one E1 (Uba2), three E2 (Ubc21, Ubc24, and UbcQ), and four E3 (Ube3-6, Ube3-7, Ube3-8, and Ube3A-2) genes were upregulated, indicating that the ubiquitin of substrates was activated under the high lead stress in *C. reinhardtii*. Cue1 recruited E2 to the ER membrane (Hirsch et al., 2009), and Usa1 could regulate the stability and/or oligomerization of E3 and recruited Der1 to E3 (Carroll and Hampton, 2010). However, blast search failed to find homolog of these two genes in *C. reinhardtii* genome, suggesting that some alternative genes exist but not yet known are functionally similar to these two genes. The last step is the extraction, processing, delivery, and degradation of ERAD substrates (Figure 6; Liu and Li, 2014). (CDC48)_6_-Npl4-ATPase-Ufd1 complex participated in the extraction of incomplete/misfolded proteins from ER lumen. Ubx was used to recruit (CDC48)_6_-Npl4-ATPase-Ufd1 complex to Hrd1/Doa10 E3 complexes on the ER membrane. After the extraction, ERAD proteins were further processed by Ufd2/3-Otu1 complex. Ufd2 is a U-box-containing E4 multiubiquitination enzyme. Otu1 is a deubiquitylating enzyme. Finally, the processed ERAD proteins were delivered to the cytosolic proteasome through Cdc48-Rad23-Dsk2 complex for the final degradation. In this study, Ufd1-2 gene in extraction, Ubx1-1 and Ubx1-2 genes in recruitment, Ufd2 and Otu1 genes in processing, and Ubp gene in degradation were all upregulated under the high lead stress. The upregulated expression of genes in the ERAD pathway suggested that the ERAD pathway was activated under the Pb stress. It is very likely that Pb stress decreased the efficiency of protein folding and increased the accumulation of incomplete/misfolded proteins in the ER, which required a highly efficient and activated ERAD pathway to remove the proteins to maintain ER homeostasis (Liu and Li, 2014). In conclusion, these changes in molecular level explained the significantly reduced content of protein under the high lead stress.

## Data Availability Statement

The datasets presented in this study can be found in online repositories. The names of the repository/repositories and accession number(s) can be found below: https://www.frontiersin.org/articles/10.3389/fmicb.2020.01443/full#supplementary-material. Sequence reads are available by the NCBI sequence read archives [SRR10269729 (control), SRR10269730 (control), SRR10269727 (low lead stress), SRR10269728 (low lead stress), SRR10269731 (high lead stress), and SRR10269732 (high lead stress)].

## Author Contributions

XL and HD designed the experiments and supervised the project. XL, CZ, XX, MA, WW, and YY performed the experiments. XL, HD, and MA wrote the original draft of the manuscript. All authors discussed the results and implications and commented on the manuscript at all stages.

## Conflict of Interest

The authors declare that the research was conducted in the absence of any commercial or financial relationships that could be construed as a potential conflict of interest.

## Footnotes

1 https://phytozome.jgi.doe.gov/pz/portal.html

2 http://pfam.xfam.org/

3 http://smart.embl.de/

4 https://www.ncbi.nlm.nih.gov/cdd/

5 http://plants.ensembl.org/index.html

6 http://meme.nbcr.net/meme3/mme.html

7 http://www.ebi.ac.uk/interpro

8 http://topcons.cbr.su.se/

9 http://www.cbs.dtu.dk/services/TMHMM-2.0/

10 http://dgpred.cbr.su.se/index.php?p=fullscan

11 http://www.cbs.dtu.dk/services/SignalP-3.0/

12 https://open.predictprotein.org/about
