# Supplementary figures and images for: Endoplasmic Reticulum-Mediated Protein Quality Control and Endoplasmic Reticulum-Associated Degradation Pathway Explain the Reduction of N-glycoprotein Level Under the Lead Stress
Source: Front Plant Sci. 2021 Jan 13;11:598552. doi: 10.3389/fpls.2020.598552 (PMC7838096; doi:10.3389/fpls.2020.598552)

Supplementary Material


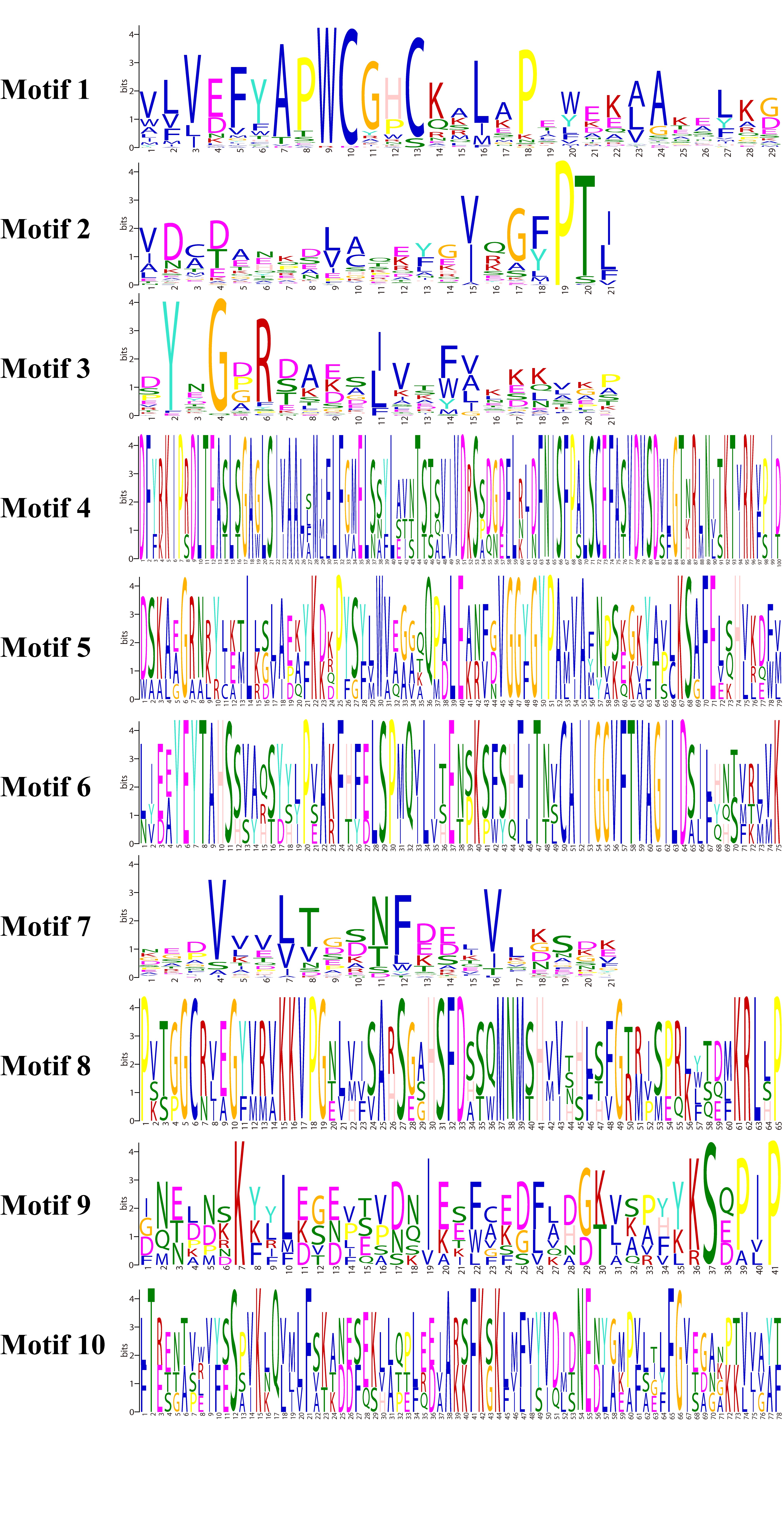


**Supplementary Figure 1.** Ten conserved motifs of PDI proteins.

Supplement: Supplementary file 1 [file Table_1.DOCX]
